# Mesenchymal Stem Cell-Derived Extracellular Vesicles Ameliorates Hippocampal Synaptic Impairment after Transient Global Ischemia

**DOI:** 10.3389/fncel.2017.00205

**Published:** 2017-07-17

**Authors:** Mingyang Deng, Han Xiao, Hainan Zhang, Hongling Peng, Huan Yuan, Yunxiao Xu, Guangsen Zhang, Zhiping Hu

**Affiliations:** ^1^Department of Hematology, The Second Xiangya Hospital, Central South University Changsha, China; ^2^Department of Neurology, The Second Xiangya Hospital, Central South University Changsha, China

**Keywords:** ischemia, mesenchymal stem cell, extracellular vesicle, COX-2, synaptic plasticity

## Abstract

Recent studies have found that administration of stem cells or extracellular vehicles (EVs) derived from stem cells exert neuroprotective effects after transient global ischemia. However, the underlying mechanisms of this effect remain unclear, especially at the level of synaptic functions. In this study, we compared the suppressive effects on cyclooxygenase-2 (COX-2) upregulation by EVs derived from bone marrow mesenchymal stem cells (BMSC-EV), adipose tissue MSC (AdMSC-EV) and serum (serum-EV). Then we examined whether BMSC-EVs could restore functional integrity of synaptic transmission and plasticity. Mice were randomly assigned to four groups: sham, sham with EV treatment, ischemia and ischemia with EV treatment. EVs were administered by intracerebroventricular injection (ICVI). We examined the consequence of transient global ischemia on pre- and post-synaptic functions of the hippocampal CA3-CA1 synapses at basal level, and long-term potentiation (LTP), an activity-dependent form of synaptic plasticity. Then we tested the therapeutic effects of EVs on these synaptic deficits. Meanwhile, Morris water maze (MWM) test was performed to examine the efficacy of EVs in rescuing ischemia-induced impairments in spatial learning and memory. EV treatment significantly restored impaired basal synaptic transmission and synaptic plasticity, and improved spatial learning and memory compared with the control group. In addition, EVs significantly inhibited ischemia-induced pathogenic expression of COX-2 in the hippocampus. EVs exert ameliorating effects on synaptic functions against transient global cerebral ischemia, which may be partly attributed to suppression of COX-2 pathogenic expression.

## Introduction

Global cerebral ischemia is a clinical outcome occurring as a consequence of cerebral infarction, cardiac arrest and severe hypotension, causing deprivation of oxygen and glucose in the brain. Ischemic stroke is a major cause of morbidity and mortality worldwide, accounting for around 85% of all stroke cases (Grupke et al., 2015).

The hippocampus is a key brain region implicated in multiple higher brain functions, such as cognition and emotion. Studies on functional anatomy of the brain demonstrated that the hippocampus is a central brain region that integrates information to represent memory (Strange et al., 2014; Zeidman and Maguire, 2016). Due to the clear axonal projection and structure in the hippocampus, various forms of synaptic plasticity, a physiological correlate of memory, such as long-term potentiation (LTP) and long-term depression (LTD), have been extensively studies in this region, especially in the Schaffer collateral pathway where CA3 axons project to CA1 pyramidal neurons (Derkach et al., 2007; Bannerman et al., 2014).

Following cerebral infarction, hippocampal CA1 neurons are vulnerable to consequent loss of blood and oxygen supply to the brain in both humans and rodents (Pulsinelli and Brierley, 1979). Among a number of pathophysiological mechanisms revealed so far, post-ischemic inflammation and the formation of free radicals are thought to play pivotal roles in reperfusion-induced delayed neuronal death. Cyclooxygenase (COX)-mediated metabolism of arachidonic acid is thought to be the primary sources of reactive oxygen species in the ischemic brain (Nogawa et al., 1998). Mounting evidence showed prolonged up-regulation of cyclooxygenase-2 (COX-2) after cerebral ischemia and the administration of COX-2 selective inhibitors has been shown to reduce brain damage and prostaglandin (PG) accumulation after cerebral ischemia (Candelario-Jalil et al., 2003; Leger et al., 2016), suggesting an important role of COX-2 in neuronal deterioration after the ischemia attack.

Meanwhile, previous studies have demonstrated that systemic administration of mesenchymal stromal cells (MSCs) prevented injury and promoted functional recovery and in the preterm brain after global hypoxia-ischemia (Jellema et al., 2013). But the underlying mechanisms remain unknown. Due to the time sensitivity of cerebral infarction in clinical cases, timely administration of stem cells cultured *in vitro* to these patients appears to be unfeasible. A recent study reported that extracellular vehicles (EVs) derived from bone marrow MSCs (BMSCs) significantly ameliorated experimental Colitis by inhibiting COX-2 expression at both mRNA and protein levels (Yang et al., 2015). Because EVs can be readily prepared from conditioned cell culture media and stored at regular conditions for a long period (e.g., 4°C for at least 1 week), therefore, administering EVs instead of MSCs may be applicable for clinical treatment.

Thus, the aim of this study was to utilize a combination of qPCR, Western blotting analysis, electrophysiological recordings and behavioral tests to assess the therapeutic potential of MSC-EVs in the regulation of neuroinflammation processes, represented as pathological COX-2 induction, and examine at functional levels, i.e., synaptic plasticity and learning and memory.

## Materials and Methods

### Animals

C57BL/6J mice were bred and group-housed in the Second Xiangya Hospital, Central South University animal facility in a 12 h-light/12 h-dark cycle (12L:12D; light intensity ~360 lux) and provided food and water *ad libitum*. Male mice at 3–4 months of age were used for *in vivo* studies. The protocol was approved by the Committee on the Ethics of the Second Xiangya Hospital, Central South University.

### Cultivation of MSC

All procedures were performed aseptically. For adipose tissue mesenchymal stem cells (AdMSC), mouse abdominal adipose tissue was minced by scalpels, washed with Hanks’ balanced salt solution (HBSS; Invitrogen, Carlsbad, CA, USA) containing antibiotics (100 IU/ml penicillin and 100 IU/ml streptomycin, Invitrogen, USA) and 2.5 μg/ml amphotericin B (Invitrogen, USA). Washed adipose tissue was digested for 2 h on a shaker at 37°C in HBSS containing 0.2% collagenase. Floating adipocytes were aspirated from pelleted stromal vascular fraction (SVF) cells after centrifugation at 400× *g* for 10 min. Pellets were suspended in red blood cell lysis buffer (2.06 g/l Tris base, 7.49 g/l NH_4_Cl, pH 7.2) for 10 min at room temperature. After suspending SVF cells in HBSS containing 2% fetal bovine serum (FBS), tissue clumps were allowed to settle for 1 min. Suspended cells were passed through 100-μm and then 40-μm cell sieves. Cell suspensions were applied to Histopaque-1077 gradients in 50-ml tubes. After centrifugation (400× *g*, 30 min), cells at the gradient interface were collected, washed in HBSS, and passed through a 30-μm mesh. For BMSC, bone marrow cell suspension was obtained by flushing marrow cavity using dispensable 1 ml syringe with low glucose DMEM, and filtrated through 200-mesh sieve to remove bone debris. The obtained bone marrow cells were shifted into culture dishes and incubated at 37°C in a humidified atmosphere containing 5% CO_2_, with low-glucose DMEM plus 10% heat-inactivated FBS and penicillin–streptomycin (Invitrogen, USA). Osteogenic and adipogenic differentiation of BMSCs was accomplished using commercially available standard differentiation induction media, namely STEMPRO^®^ osteogenesis differentiation kit and STEMPRO^®^ adipogenesis differentiation kit (Invitrogen) in accordance with the manufacturer’s instruction. Differentiation potential was assessed by histological alizarin red staining for osteogenic differentiation and oil-red-O staining for adipogenic differentiation at day 28 of differentiation induction. The absence of hematopoietic markers CD45 and CD11b on these BMSC cells were confirmed by flow cytometry analysis. Briefly, DMSCs were washed twice in FACS medium phosphate buffered PBS containing 1% FCS and 0.1% NaN3. Then the cells were incubated for 30 min at 4°C with CD45 and CD11b antibodies (Abcam, Cambridge, MA, USA) according to the standard procedure. An isotype control was used for each antibody. Fluorescence was measured by using a FACSCalibur (Becton Dickinson, San Diego, CA, USA) and data were analyzed by using the CellQuest Software (Becton Dickinson, San Diego, CA, USA). Results are shown in Supplementary Figure S1.

### Purification of EVs

When AdMSC and BMSC grew to the 4th to 6th passages at 70%–80% confluence, cells were washed with PBS for three times and then incubated DMEM supplemented with 10% exosome-depleted FBS (SBI, USA). Forty-eight hours later, EVs were collected with ExoQuick-TC kit (SBI, USA) from conditioned media, following manufacturer’s manual description. Serum-EVs were purified from mouse serum using ExoQuick kit (SBI, USA). Briefly, mouse tail blood was collected into microcentrifugation tubes, sit at 37°C for 30 min and refrigerated at 4°C for 2 h, and then were centrifuged at 1600 *g* for 10 min. Clear serum was transferred to fresh tubes and centrifuged at 10,000 *g* for another 10 min at 4°C to remove cells and debris. Next, we mixed 250 μL of centrifuged serum with 63 μL of ExoQuick reagent. The samples were incubated at 4°C for 30 min and then centrifuged at room temperature at 1500 *g* for 30 min. The supernatant was discarded and the EV pellet was suspended in 100 μL of PBS. The EV suspension was used for further examination. The amount of EVs was measured by total EV-associated proteins using the Bradford protein assay (Beyotime Biotechnology, Shanghai, China). We typically obtain EVs containing 100–200 μg protein from 8 ml medium per 10-cm plate of BMSC or AdMSC at confluency (4 × 10^6^ cells) conditioned for 48 h. The yield of EVs from mouse serum is typically 5 mg EV protein corresponding to 1 ml serum. For EVs from cultures, we pooled EVs from 6 to 8 10-cm plates (8 ml media from each plate) together as one batch. For EVs from serum, we pooled EVs from two mice as one batch. The purity of EVs were examined by Western blot using positive markers Alix (Abcam) and CD63 (Abcam), and a negative marker GM130 (Abcam). Results are shown in Supplementary Figure S2.

### Cortical Neuron-Glia Culture

Neuron-glia cultures were prepared from the cerebral cortices of embryonic day 17 C57BL/6J pups. All procedures were performed aseptically. The meninges were removed and dissociated by trituration in 0.25% pre-warmed trypsin. After trypsinization, cells were harvested and seeded at a density of 5 × 10^5^ cells in 6-well plates pre-coated with 10 μg/ml of poly-D-lysine (Sigma, St. Louis, MO, USA). The culture medium consisted of MEM supplemented with 2% FBS, 1 *g*/L glucose, 2 mM L-glutamine, 1 mM sodium pyruvate, 100 mM nonessential amino acids and antibiotics. Cultures were maintained in a humidified 5% CO_2_ incubator at 37°C, and were used for the experiment at DIV18–20.

### Electrophysiology of Field Recordings

Acute hippocampal slices were prepared 1 day after ischemia or sham operation. Mice were anesthetized with isoflurane and sacrificed by decapitation. Whole brains were rapidly removed and placed in an ice-cold cutting solution containing/consisting of (in mM): 234 sucrose, 2.5 KCl, 1.25 NaH_2_PO_4_, 10.0 MgSO_4_, 1 CaCl_2_, 26 NaHCO_3_ and 20 glucose, saturated with 95% O_2_ and 5% CO_2_. After 5 min incubation in the ice-cold sucrose solution, hippocampi were removed and glued on the stage of VIBRATOME VT 3000 (Leica) and immersed in ice-cold cutting solution (50%) and normal external (50%) solution. Coronal slices (400 μm thick) were cut and transferred to normal external solution containing (in mM): 124 NaCl, 2.5 KCl, 2.5 CaCl_2_, 1.3 MgSO_4_, 26 NaHCO_3_, 1 NaH_2_PO_4_ and 10 glucose. All solutions were saturated with 95% O_2_ and 5% CO_2_ (pH 7.4). Slices were incubated for at least 1 h in the external recording solution at 28 ± 1°C prior to recording. Slices were transferred to a submersion-type recording chamber mounted on the stage of an upright microscope (BX50WI, Olympus, Tokyo, Japan), held fixed by a grid of parallel nylon threads and perfused with external solution at a rate of 2 ml/min. A patch electrode (1–2 MΩ) filled with external solution was positioned in the stratum radiatum of area CA1. Field excitatory post-synaptic potentials (fEPSPs) were evoked by square pulses in Schaffer collateral afferents by means of a bipolar tungsten stimulating electrode (FHC, Bowdoin, ME, USA). Stimulations were generated with a pulse generator (Grass-88X, USA). Baseline pre-synaptic stimulation was delivered once every 20 s using a stimulation intensity yielding 40%–60% of the maximal response (for LTP experiments). The initial slope of the fEPSP was used to measure stability of synaptic responses and to quantify the magnitude of LTP. For input/output (I/O) curves, single-pulse monophasic test stimulation was applied at 0.033 Hz, and the electrode positioning was adjusted to maximize amplitude of the fEPSP. After stable baseline recording for at least 20 min, LTP was induced by delivering two trains of stimuli (two trains of 100 pulses at 100 Hz separated by 20 s). Field potentials were acquired using a Multiclamp 700B amplifier (Axon Instruments, USA) and digitizer 1440 (Axon Instruments, USA).

### Western Blot Analysis

Tissues were lysed in a buffer containing 20 mM Tris-HCl (pH 7.6), 250 mM NaCl, 0.5% NP-40, 3 mM EDTA and 1.5 mM EGTA with 10 μg/ml Aprotinin, 10 μg/ml leupeptin, 1 mM DTT, 1 mM PNPP and 0.1 mM Na_3_VO_4_ as protease and phosphatase inhibitor. After centrifugation, lysates (100 μg/lane) were subjected to 10% SDS-PAGE and transferred onto polyvinylidene difluoride membranes (Roche, Germany). The membranes were blocked for 1 h in TBST (25 mM Tris-HCl, pH 7.6, 125 mM NaCl, 0.1% Tween-20) containing 5% nonfat dried milk, and then the membrane was incubated with antibodies was diluted in TBST containing 5% nonfat dried milk at 4°C overnight. The primary antibodies include COX-2 antibody (1:400, Abcam), β-tubulin (1:500, Sigma), Alix (1:1000, Abcam), CD63 (1:1000, Abcam), GM130 (1:500, Sigma). HRP conjugated secondary antibodies were purchased from Sigma. Protein bands were detected by the Immobilon Western Chemiluminescent HRP Substrate (Millipore, USA) and images were taken by FluorChem FC2 System (Alpha Innotech Corporation, USA).

### qRT-PCR

Total RNA from tissues was extracted using TRIzol reagent and RNA prepared using Qiagen RNEasy Midi Kit. Total RNA (1 μg) was treated with TURBO DNase and reverse transcribed using random hexamers and the Superscript III First-Strand Synthesis System for RT-PCR according to the manufacturer’s instructions. Negative controls (no first-strand synthesis) were prepared by performing reverse transcription reactions in the absence of reverse transcriptase. PCR amplification was performed with Platinum SYBR Green qPCR SuperMix UDG. Reactions were performed in duplicate, and contained 2× SYBR Green qPCR SuperMix, 1 μl of template cDNA or control, and 100 nM primers (COX-2, forward: TGAGCAACTATTCCAAACCAGC; reverse: GCACGTAGTCTTCGATCACTATC) in a final volume of 25 μl and analyzed in 96-well optical reaction plates (Applied Biosystems). Reactions were amplified and quantified using an Applied Biosystems 7700 sequence detector with standard cycle conditions and the Applied Biosystems software. Relative quantities of mRNA in duplicate samples were obtained using the ΔΔCT method and normalized against mouse tubulin as endogenous controls.

### Transient Global Cerebral Ischemia and EV Injection

Mice were anesthetized with a mixture of 2.5% isoflurane in 33% oxygen and 67% nitrous oxide. Bilateral common carotid arteries were isolated and occluded using non-traumatic aneurysm clips. The complete interruption of blood flow was confirmed by observing the central artery in retina using an ophthalmoscope. After 5 min of occlusion, the aneurysm clips were removed from the common carotid arteries. The rectal temperature was monitored and maintained using a thermometric blanket before, during and after the surgery until the animals completely recovered from anesthesia. Thereafter, animals were kept on the thermal incubator to maintain the body temperature of animals until the animals were euthanized. Sham-operated animals were subjected to the same surgical procedures except that the common carotid arteries were not occluded. Intracerebroventricular injections (ICVI) of 200 μg EVs dissolved in phosphate-buffered saline (PBS) into the cerebral ventricle were made according to stereotaxic coordinates PA-1.0 mm, lateral-1.5 mm from bregma, and ventral-2.0 mm relative to dura.

### Morris Water Maze

Hippocampus-dependent spatial learning and memory abilities were evaluated with the Morris water maze (MWM) test. Briefly, the mouse water maze was divided into four quadrants. A hidden platform was placed 2 cm below the water surface in the center of one quadrant during training. The mice were subjected to training trials (the navigation test), followed by probing without the platform. During the training trials, mice were released into the maze from a randomly selected quadrant and with all animals using the same order. The escape latency to find the underwater platform was recorded. Each animal performed five training sessions from different starting quadrants per day. The escape latency (seconds) were analyzed. Data from trials in each daily session were averaged for each mouse. To assess spatial learning ability, the platform was removed from the pool, and the animals were subjected to a 60 s probe trial following the last training session to find the original platform (target crossings). The proportion of time spent in the target quadrant, were monitored and recorded by a video camera linked to a computer-based image analyzer.

### Statistics

Data was analyzed by one or two-way ANOVA analysis followed by a Tukey’s *post hoc* test or Student’s *t*-test, indicated in the figure legends. Statistical significance was determined when *p* value was less than 0.05. *N* in each experiments indicate the number of independent experiments, including independent preparation of EVs, cell culture, lysate preparation and Western blot analysis.

## Results

### EVs from Bone Marrow MSC (BMSC) Significantly Inhibit LPS-Induced COX-2 Expression

**Figure 1:**
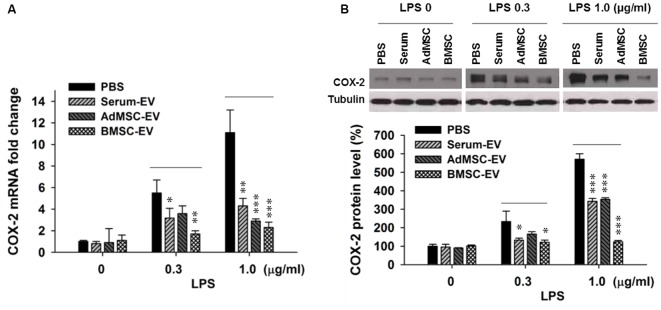
Effects of extracellular vehicles (EVs) on lipopolysaccharides (LPS-induced) cyclooxygenase-2 (COX-2) expression at mRNA and protein levels in mixed neuron-glial primary cultures. **(A)** EVs derived from serum (serum-EV), adipose mesenchymal stem cells (AdMSC-EV) and bone marrow MSC (BMSC-EV) significantly suppressed LPS (0.3 μg/ml and 1.0 μg/ml) -induced up-regulation of *COX-2* mRNA level, as compared to phosphate-buffered saline (PBS) treatment (LPS 0.3 μg/ml: PBS = 5.7 ± 0.6, serum-EV = 3.3 ± 0.6, AdMSC-EV = 3.8 ± 0.5, BMSC = 1.9 ± 0.2). **(B)** EVs from three sources significantly inhibited LPS-induced COX-2 at protein level. The three straight lines above sample blot images indicate the three groups of LPS treatment at the concentrations of 0, 0.3 and 1.0 μg/ml (LPS 0.3 μg/ml: PBS = 243 ± 51%, serum-EV = 132 ± 10%, AdMSC-EV = 179 ± 14%, BMSC = 127 ± 13%). *n* = 7 tests per group. Data show means ± SEM. One-way ANOVA analysis followed by a Tukey’s *post hoc* test. *p < 0.05, **p < 0.01 and ***p < 0.001.

Upregulated pathogenic expression of *COX-2* mRNA and protein has been observed in CA1 hippocampal neurons after global ischemia in animal models (Nakayama et al., 1998; Choi et al., 2006). COX-2-deficient mice exhibited less vulnerability to ischemic neuronal death (Nakayama et al., 1998). Therefore, in this study we used COX-2 expression level as an indicator of ischemic injury. It has been reported that EVs with therapeutic potential can be derived from diverse sources, such as serum (serum-EV), adipose tissue MSC (AdMSC-EV) and BMSC-EV (Lin et al., 2016; Dostert et al., 2017; Phinney and Pittenger, 2017; Toh et al., 2017; Yang et al., 2017). To determine EVs derived from which source are more effective to inhibit the expression of major pro-inflammation factor COX-2, after the examination of the purity of these EVs (Supplementary Figure S1), we performed *in vitro* assay that three types of EVs were added in primary cortical neuron-glia cultures prepared from mouse cortices, right after the addition of lipopolysaccharides (LPS). LPS (lipoglycan or endotoxin), consisting of a lipid and a polysaccharide composed of O-antigen, is found in the outer membrane of Gram-negative bacteria, and commonly used to elicit immune responses *in vivo* and *in vitro*. Twenty-four hours later, cells were collected for qPCR and Western blotting analysis. Figure 1A shows that the addition of LPS at both 0.3 μg/ml and 1.0 μg/ml drastically induced the transcription of *COX*-*2* mRNA. One-hundred microgram per ml of serum-EV and BMSC-EV both significantly prevented this increase of COX-2 mRNA induced by 0.3 μg/ml LPS treatment, and EVs derived from all three types of resources largely inhibited *COX-2* mRNA up-regulation after 1.0 μg/ml LPS treatment. Consistent with changes at mRNA levels (Figure 1A), the increase of COX-2 immunoreactivity was also significantly prevented by serum-EV, AdMSC-EV and BMSC-EV (Figure 1B). Importantly, note that BMSC-EV, among three types of EVs, exhibited the strongest effects of suppressing COX-2 induction at both mRNA and protein levels. After we characterized BMSCs by testing their differentiation potentials and the absence of hematopoietic markers CD45 and CD11b (Supplementary Figure S2), we prepared BMSC-EVs for further experiments.

### BMSC-EV Significantly Inhibit COX-2 Expression Induced by Transient Global Cerebral Ischemia

**Figure 2:**
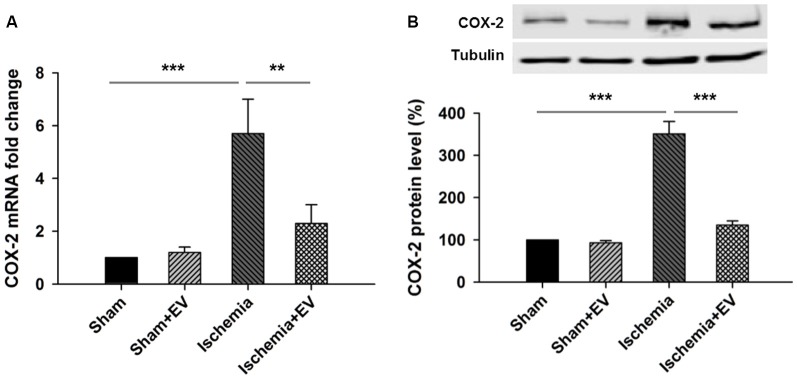
Effects of BMSC-EVs on 5 min of bilateral common carotid artery occlusion-induced COX-2 expression at mRNA and protein levels in the hippocampi. Intracerebroventricular injection (ICVI) of 200 μg BMSC-EVs right before occlusion largely suppressed pathogenic expression of COX-2 at **(A)** mRNA level (sham + EV = 96 ± 4, ischemia = 5.7 ± 1.5, ischemia + EV = 2.2 ± 0.7) and **(B)** protein level (sham + EV = 1.2 ± 0.15, ischemia = 341 ± 39, ischemia + EV = 128 ± 10). *n* = 8 tests per group. Data show means ± SEM. One-way ANOVA analysis followed by a Tukey’s *post hoc* test. **p < 0.01 and ***p < 0.001.

Next, to directly test the inhibitory effects of BMSC-EVs (hereinafter referred to as EVs) on COX-2 up-regulation in ischemic brain, we performed ICVI of 200 μg EVs dissolved in PBS into the cerebral ventricle, right before transient global cerebral ischemia procedure. Twenty-four hours after the procedure, mice were sacrificed and hippocampi were quickly dissected in ice-cold PBS and snap-frozen in liquid nitrogen and stored at −80°C for future qPCR and Western blotting analysis. ICVI of EVs did not alter *COX-2* mRNA level in sham group. However, *COX-2* mRNA level in the hippocampi from the ischemia group undergone 5 min of occlusion of bilateral common carotid arteries increased about 6-folds which was largely prevented by ICVI injection right before the artery occlusion procedure (Figure 2A). Total hippocampal protein lysate prepared from the same four groups of mice also showed changes of immunoreactivity similar to that of mRNA levels (Figure 2B).

To this end, we have demonstrated that BMSC-EVs significantly prevented up-regulation of COX-2 expression induced by LPS in *in vitro* assay and by transient global cerebral ischemia procedure *in vivo*. Given that COX-2-mediated neuroinflammation has been shown to be one of the major contributing factors in the impairment of hippocampal functions in ischemic brain, these results indicate that BMSC-EVs may effectively ameliorate deficits in learning and memory induced by transient ischemia through inhibiting COX-2 expression.

### BMSC-EVs Partly Rescue Ischemia-Induced Deficits in CA1 Basal Synaptic Transmission

**Figure 3:**
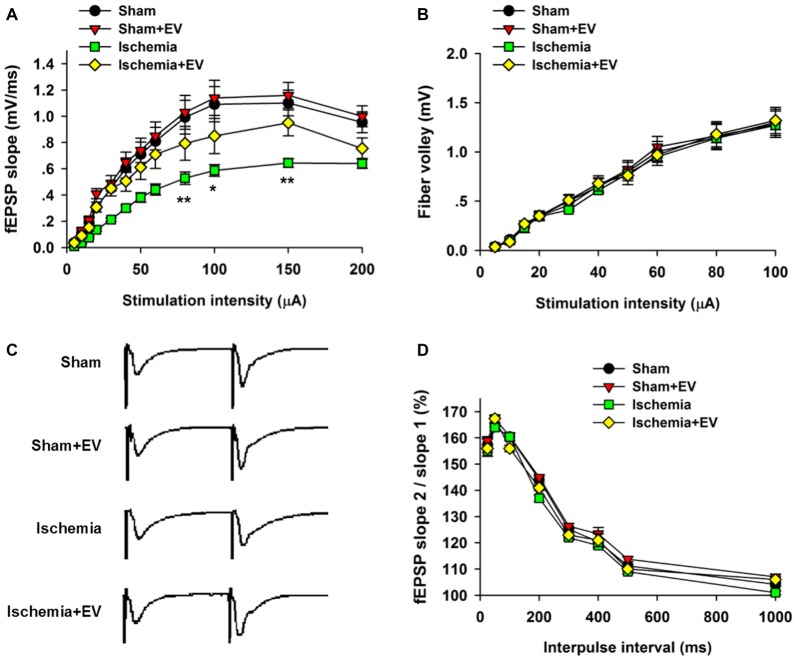
Effects of ischemia and BMSC-EVs on basal properties of excitatory synaptic transmission in animals subjected to global ischemia. **(A)** Input-output (I/O) ratios of field excitatory post-synaptic potentials (fEPSPs) slope at Schaffer collateral to CA1 synapses were recorded from stratum radiatum in acute hippocampal slices (80 μA: sham = 0.99 ± 0.13, sham + EV = 1.03 ± 0.13, ischemia = 0.53 ± 0.05, ischemia + EV = 0.80 ± 0.12; 100 μA: sham = 1.10 ± 0.14, sham + EV = 1.15 ± 0.15, ischemia = 0.59 ± 0.04, ischemia + EV = 0.85 ± 0.14; 150 μA: sham = 1.10 ± 0.12, sham + EV = 1.16 ± 0.11, ischemia = 0.65 ± 0.04, ischemia + EV = 0.95 ± 0.11). **(B)** Fiber volley amplitude were recorded as a function of stimulus intensity. **(C,D)** BMSC-EVs does not alter release probability in mice subjected to global ischemia. fEPSPs evoked by paired pulse stimulation of Schaffer collaterals at 25, 50, 100, 200, 300, 400, 500 and 1000 ms were recorded. **(C)** shows representative fEPSP traces with 100 ms of inter-pulse interval. *n* = 15 neurons from four mice per group. Data show means ± SEM. Two-way ANOVA analysis followed by a Tukey’s *post hoc* test. *p < 0.05, **p < 0.01.

Whereas BMSC-EVs have been shown to inhibit ischemia-induced COX-2 expression in Figure 2, its impact on the functional integrity of hippocampal CA3 to CA1 synaptic transmission is unclear. To address this issue, we prepared acute hippocampal slices 24 h after the transient global cerebral ischemia procedure. We focused on the synapses formed on CA1 pyramidal neurons because these neurons are critically involved in multiple forms of learning and memory, meanwhile are the most vulnerable cells in animals subjected to global ischemia (Pulsinelli and Brierley, 1979; Nakayama et al., 1998; Choi et al., 2006). First, by recording fEPSPs elicited by test pulses delivered to Schaffer collaterals at different stimulus intensities. By analyzing the slope I/O relation of fEPSPs, we found that transient ischemia greatly reduced post-synaptic responses elicited by the stimuli at the same intensities as the sham group (Figure 3A). However, EV treatment largely rescued this deficit in basal synaptic transmission. EV treatment did not show effects in the sham mice.

To examine possible changes in pre-synaptic function, we carried out two experimental paradigms. First, we monitored the fiber volley amplitude as an indicator of viability of pre-synaptic fibers at CA3-CA1 synapses. Transient global ischemia did not detectably alter the fiber volley amplitude in the four groups of mice (Figure 3B). Paired-pulse ratio (PPR) is a form of short-term plasticity commonly used as an indicator of probability of pre-synaptic transmitter release (Blitz et al., 2004). Similar to the fiber volley amplitude, EV treatment did not alter the PPR at CA3-CA1 synapses of mice subjected to global ischemia (Figures 3C,D). These results suggest that EV treatment does not alter pre-synaptic functions, but significantly rescues post-synaptic impairment of synaptic transmission.

### BMSC-EVs Partly Restore Impairment in Long-Term Potentiation (LTP)

**Figure 4:**
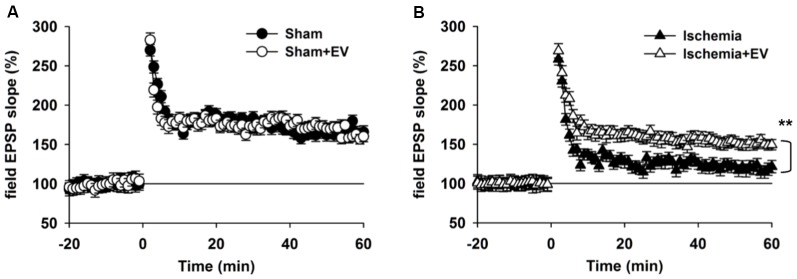
BMSC-EVs rescues impaired hippocampal long-term potentiation (LTP) in mice subjected to global ischemia. **(A)** LTP was induced by high frequency stimulation (HFS, two trains at 100 Hz for 1 s, separated by 10 s) in acute hippocampal slices prepared from mice 1 day after surgery. **(A)** EV treatment did not notably alter LTP in sham-operated mice (mean of the last 10 min: sham = 165 ± 8.1% and sham + EV = 162 ± 6.9%). **(B)** Global ischemia largely impaired LTP 1 day days after reperfusion. EV treatment significantly prevented the reduction of the mean amplitude of the last 10 min of the recordings (ischemia = 121 ± 8.3% and ischemia + EV = 162 ± 7.9%). *n* = 10 slices from 5 mice per group. Data show means ± SEM. Student’s *t*-test. **p < 0.01.

Hippocampal synaptic plasticity, such as LTP, has been extensively studied and is presently the most widely accepted cellular and molecular model of learning and memory. Post-synaptic α-amino-3-hydroxy-5-methyl-4-isoxazolepropionic acid (AMPA) type glutamate receptor (AMPAR) insertion is thought to be the underlying mechanism of LTP. Our data in Figure 3 strongly indicate a functional role of EV treatment in post-synaptic events. To further explore this issue, we applied high frequency stimulation (HFS) to induce N-methyl-D-aspartate receptor (NMDAR)-dependent LTP at CA3-CA1 pyramidal cell synapses. As shown in Figure 4A, EV treatment did not affect LTP amplitude up to 60 min post HFS. However, EV significantly restored impaired LTP in the ischemia group (Figure 4B). These results strongly suggest that EVs act at post-synaptic sites to ameliorate impaired hippocampal functions.

### BMSC-EVs Ameliorate Performance of Ischemic Mice in Morris Water Maze Test

**Figure 5:**
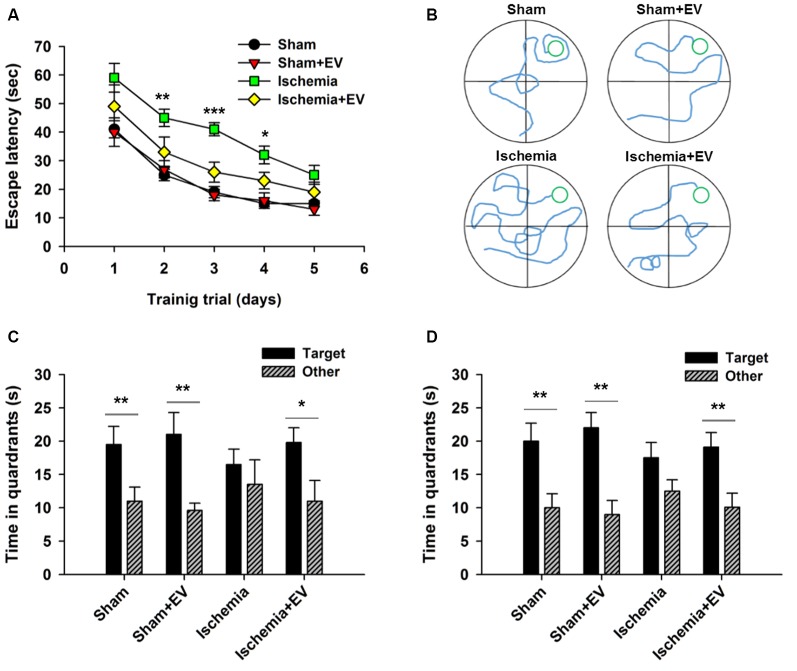
Morris water maze (MWM) test of spatial learning and memory in BMSC-EV treated ischemic mice. **(A)** Escape latency measured as mean time (s) during training trials (day 2: sham = 25.1 ± 2.1, sham + EV = 26.8 ± 3.3, ischemia = 44.9 ± 3.1, ischemia + EV = 33.5 ± 5.3; day 3: sham = 18.7 ± 2.0, sham + EV = 17.6 ± 2.5, ischemia = 41.1 ± 2.3, ischemia + EV = 16.3 ± 3.5; day 4: sham = 14.8 ± 1.3, sham + EV = 16.8 ± 2.3, ischemia = 31.9 ± 3.1, ischemia + EV = 23.0 ± 2.8). **(B)** Representative swimming traces of the four groups of mice exhibited the training trial on day 3. **(C)** Time spent in target quadrant and other quadrants 24 h after the last training trial (sham: 19.5 ± 2.7 and 11.0 ± 2.1; sham + EV: 21.4 ± 3.6 and 9.6 ± 1.1; ischemia: 16.6 ± 2.4 and 13.5 ± 3.7; ischemia + EV: 19.8 ± 2.2 and 10.9 ± 3.1). **(D)** Time spent in target quadrant and other quadrants tested 7 days later (sham: 19.8 ± 2.6 and 11.6.0 ± 2.1; sham + EV: 22.3 ± 2.3 and 9.1 ± 1.9; ischemia: 17.5 ± 2.4 and 12.3 ± 1.8; ischemia + EV: 19.1 ± 2.5 and 10.8 ± 3.1). *n* = 12 per group. Data show means ± SEM. Two-way ANOVA analysis followed by a Tukey’s *post hoc* test for **(A)**. Student’s *t*-test for **(C,D)**. *p < 0.05, **p < 0.01 and ***p < 0.001.

MWM is commonly used to assess animals’ spatial learning and memory that requires the integrality the hippocampus (Morris, 1984; Nakazawa et al., 2004). Figure 5A shows the mean latency for mice finding the hidden platform (escape latency) during the place navigation test. Analysis showed a progressive reduction over successive training trials lasting for 5 days. Ischemia groups required longer time searching for the platform, compared to the sham-operated group. EV treatment significantly shortened this time. Figure 5B shows representative swimming traces of the four groups of mice exhibited in the training trial on day 3. Twenty-four hours after the last training trial, we carried out a probe test showing that mice from sham, sham + EV and ischemia + EV groups, but not from the ischemia group, spent significantly more time swimming in the target quadrant (Figure 5C), suggesting alleviation of impaired short-term memory in ischemia brain by EV treatment. Seven days later, the same groups of mice were probed again, as an assessment for long-term memory. Similar to the test for short-term memory, mice from sham, sham + EV and ischemia + EV groups, but not from the ischemia group, spent significantly more time swimming in the target quadrant searching for the platform (Figure 5D).

## Discussion

In summary, this study first compared the inhibitory effects of EVs prepared from three different sources (serum, AdMSC and BMSC) on LPS-induced COX-2 up-regulation in mixed neuronal-glia cultures. BMSC-EVs showed the strongest effects therefore used in the following *in vivo* studies. ICVI of 200 μg EVs right before bilateral common carotid artery occlusion significantly prevented ischemia-induced COX-2 pathogenic expression at both mRNA and protein levels. Functional analysis using electrophysiological recordings on acute hippocampal slices prepared 24 h after the surgery revealed that transient global ischemia mainly affects post-synaptic events, as evidenced by impaired I/O ratio of fEPSP slope, but unaltered viability of pre-synaptic fibers or transmitter release probability. This impairment of basal synaptic transmission was partly rescued by EV treatment. Post-synaptic AMPAR insertion is thought to underlie LTP. Our recordings showed that ischemia markedly impaired LTP amplitude, which can be partly restored by BMSC-EVs. In behavioral tests, mice in ischemia group exhibit significantly longer escape latency during training trials in Marris water maze tests, suggesting deficits in spatial learning, a brain function critically requires the integrity of the hippocampus. EV treatment partly rescued this deficit. Later, in both short-term and long-term memory retrieval tests, EV treatment showed significant ameliorating effects on memory impairments. Together, these data suggest that EVs derived from bone marrow stem cells (BMSC-EV) can effectively ameliorate impairments of hippocampal spatial learning and memory induced by transient cerebral globe ischemia, possibly through inhibition of COX-2 pathological expression. However, we cannot rule out the possibility that the reduced COX-2 expression may be a consequence of reduced pathological injury. Co-administration of a COX-2 inhibitor may be applied in future studies to clarify the causal-consequential relation between increased COX-2 level and pathological injury. Since EVs are more readily prepared, stored and administered than therapeutic stem cells, our study shed light on therapeutic potential of EVs in clinical applications. However, there are caveats related to the fact that the source cells (e.g., patient specific autologous MSCs), may still need to be isolated and extensively expanded to derive enough EVs for clinical applications. One possibility to circumvent this issue could be administering previously isolated allogenic/aploidentical EVs to acute stroke patients. Another issue requires further exploration is the delivery routes of EVs. The ICVI method used in this study ensures the successful delivery of EVs into the mouse brain, but it is not applicable to treat patients. The efficiency and efficacy of EVs reaching the site of ischemia via intravenous and intranasal administration remain to be tested.

The differential inhibitory efficacy of EVs from three sources on COX-2 expression may be explained by different immune modulatory properties of MSCs *per se*, and the possibility that these EVs may exert effects on different pro-inflammatory factors. For example, two of the most prominent pro-inflammatory factors drastically induced by ischemia are nitrogen monoxide (NO) by nitric oxide synthase (NOS) and the formation of PGs by COX-2 (Nogawa et al., 1998; Tabassum et al., 2015; Takeuchi et al., 2017). In this study we used COX-2 expression level as the indicator of ischemic insults to the hippocampus throughout the study. Previous studies have shown that in liver and kidney ischemia-reperfusion models, exosome treatment broadly suppressed pro-inflammatory factors including Matrix Metalloproteinase-9, Interleukin-1β, Tumor Necrosis Factor -α, COX-2 and oxidative stress markers (NADPH oxidase 1 and 2) and apoptosis markers (cleaved caspase 3 and PARP), DNA damage (γ-H2AX) and mitochondrial damage (cytosolic cytochrome-C) markers (Lin et al., 2016; Sun et al., 2017). Therefore, COX-2 is one of many downstream effectors of EV treatment. Given the complexity of the contents in EVs, the exact regulating pathway of COX-2 upregulation remains elusive. Moreover, it is noteworthy that although studies show that COX-2 activity contributes to CA1 neuronal death after global ischemia (Nakayama et al., 1998; Takeuchi et al., 2017), it may also contribute to the recovery of neural functions by enhancing the proliferation of neural progenitor cells after ischemia (Sasaki et al., 2003). It is possible that COX-2 plays differential roles at different stages of the pathogenesis after ischemia induction. Another question to be addressed in future studies is the identification of the type of cells of COX-2 expression under transient global ischemia condition, and whether EVs exert neuroprotective effects through modulating the source cells of COX-2 or directly on neurons. These results would provide a deeper insight into the underlying mechanisms of EVs’ treatment effects. In addition, the initial screening approach in this study used LPS-induced upregulation of COX-2 in cell cultures as the indicator to test differential suppressive effects of EVs derived from different sources. This result may not represent the COX-2 upregulation induced by transient global ischemia in live animals. Although we did observe significant therapeutic effects of BMSC-EVs on impaired neural functions, we cannot conclude that the AdMSC-EVs or serum-EVs may exhibit comparable ameliorating effects under *in vivo* conditions.

Global ischemia in humans induced experimentally in animals causes selective, delayed neuronal death in pyramidal neurons of the hippocampal CA1 (Pulsinelli and Brierley, 1979; Nakayama et al., 1998). However, to our knowledge, our study is the first report providing electrophysiological recording results showing synaptic mechanisms of functional rescue by MSC-EVs treatment. Unchanged fiber volley and PPR are in line with the conserved viability of the presynaptic fibers which project from neurons in the CA3 subfield, known to resist transient global ischemia *in vivo* (Liou et al., 2003; Moskowitz et al., 2010; Ofengeim et al., 2012). Moreover, partial rescue of basal excitatory synaptic transmission and synaptic plasticity in the form of LTP at Shaffer-collateral to CA1 synapses reveals a previously unknown role of EVs in preserving the synaptic functions of CA1 neurons. Consistent with electrophysiological data, MWM results showed that ischemic mice spent more time searching for the hidden platform during the training trials and less time in the target quadrant in the memory tests. These deficits were attenuated by EV treatment, but we could not conclude from these data that the cause of memory deficits was attributable to impaired learning process or memory retrieval process.

Previous studies using stem cell transplantation demonstrated that MSC transplantation may be effective in neuroprotection after ischemia, however, only a small proportion of administered MSCs were physically located into injured tissues (Rosario et al., 1997), suggesting that the ameliorating effects of these cells may be attributable to paracrine activity of MSCs, such as the EVs. EVs has been demonstrated to protect injuries in various conditions, including acute kidney injury (Ju et al., 2015), vascular injury (Jansen et al., 2013) and pulmonary hypertension (Lee et al., 2012). Inhibiting neuroinflammation as the underlying mechanism of MSC-EVs therapy is supported by several studies showing beneficial effects of MSC-EV treatment after ischemia (Baglio et al., 2012; Pan et al., 2014).

This study proposes potential therapeutic application of EVs offering several advantages over the administration of MSCs: first, EV application avoids the risk for possible malignant transformation of MSCs; second, EVs can better penetrate into tissues of injury due to its small size; third, the production and the quality control processes of EVs for clinical application are more feasible than stem cell therapy. However, although we did not observe adverse effects in the sham control group following the EV treatment in this study, future introduction of MSC-EVs into clinical trials warrants further in-depth analyses of adverse effects. In addition, it is necessary to point out, in this study, we administered EVs immediately prior to the instruction of transient global ischemia, so that the ameliorating effects of EVs could take place from the beginning of ischemia-induced neural impairments. Since the unset of ischemia attacks are unpredictable in clinical scenario, future studies are warranted to determine the time window of therapeutical effectiveness of EV administration after the onset of ischemia.

## Conclusion

In summary, we present results showing that EVs derived from serum, AdMSC and BMSC manifested inhibitory effects on pathogenic COX-2 expression. Administration of BMSC-EVs right before transient ischemia induction significantly inhibited COX-2 expression in the hippocampi and partially rescued the synaptic transmission and plasticity of the CA1 pyramidal neurons, and spatial learning and memory. This study highlights therapeutic potential of EVs in clinical applications.

## Author Contributions

MD and HX conceived and designed experiments; HX provided reagents; MD, HZ, HP, HY, YX, GZ and ZH performed experiments; MD and HZ analyzed the data; HX wrote the article.

## Conflict of Interest Statement

The authors declare that the research was conducted in the absence of any commercial or financial relationships that could be construed as a potential conflict of interest. The reviewer JH and handling Editor declared their shared affiliation, and the handling Editor states that the process nevertheless met the standards of a fair and objective review.
