# Supplementary material for: Mesenchymal Stem Cell-Derived Extracellular Vesicles Ameliorates Hippocampal Synaptic Impairment after Transient Global Ischemia
Source: Front Cell Neurosci. 2017 Jul 17;11:205. doi: 10.3389/fncel.2017.00205 (PMC5511812; doi:10.3389/fncel.2017.00205)
Supplement: Supplementary file 1 [file Presentation_1.pdf]

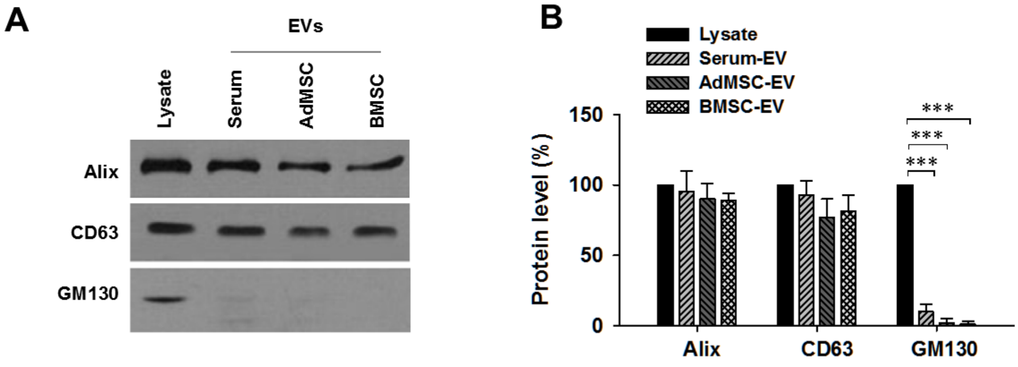

Figure S1. Characterization of EVs by Western blot probed with positive markers Alix and CD63, and negative marker GM130. (A) Sample images of Alix, CD63 and GM130 protein levels of EVs derived from serum (serum-EV), adipose MSC (AdMSC-EV) and bone marrow MSC (BMSC-EV). 293T cell lysate was used as the positive protein control. 15 μg protein samples were loaded in each lane. (B) Statistic results showing GM130 was significantly lower in EVs (serum-EV=10.1±4.9%, AdMSC-EV=2.3±1.0%, BMSC=1.8±1.3%). n=4 tests per group. Data show means±SEM. Student's t-test. ***$p<0.001$

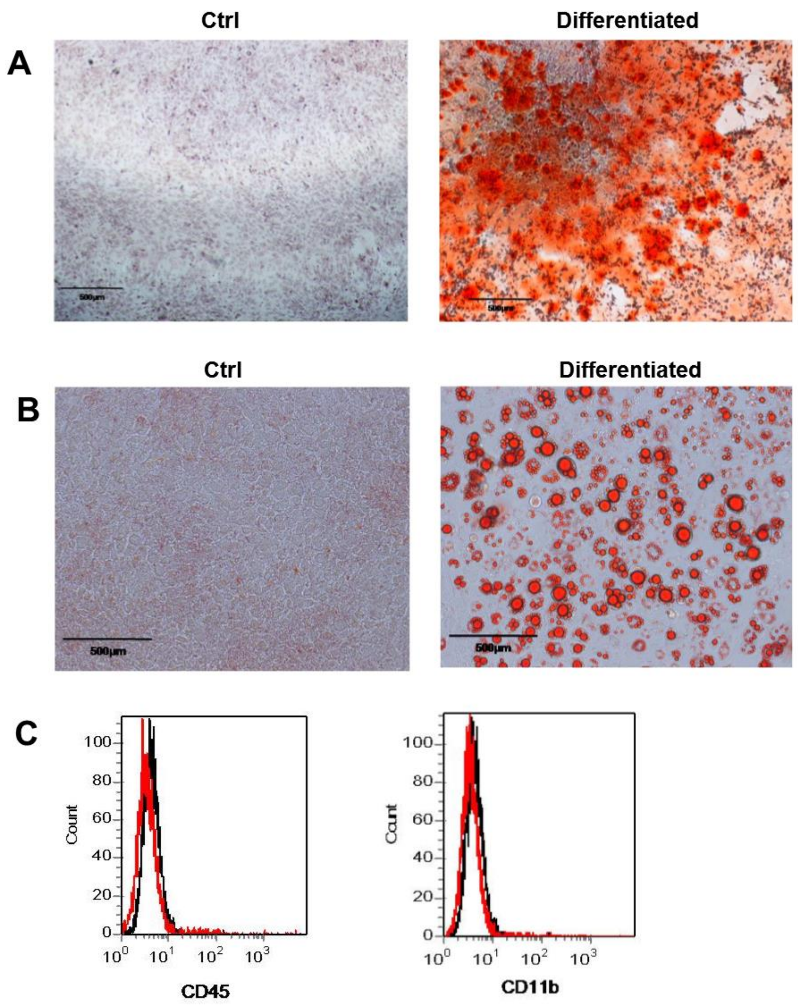

Figure S2: Characterization of BMSC cells. (A) Osteogenic differentiation of BMSC cultured in standard MSC growth medium (control, left panel) or osteogenic induction medium (differentiated, right panel) for 28 days, revealed by alizarin red staining. Scale bar: 500 µm. (B) Adipogenic differentiation assay of BMSC cultured in standard MSC growth medium (control) or adipogenic induction medium (differentiated) for 28 days, revealed by staining with oil-red-O staining. Scale bar: 500 µm. (C) Flow cytometry assay histogram of BMSC cells expressing CD45 and CD11b. The BMSCs were gated fall within established viable cell forward and side scatter parameters and to eliminate the cell aggregates. The surface expression of CD45 and CD11b were analyzed in a FL2 histogram. Data are reported at positive cells.
